# DAILY EMOTION EXPRESSION, MODES OF SOCIAL CONTACT, AND WELL-BEING IN LATE LIFE

**DOI:** 10.1093/geroni/igae098.3228

**Published:** 2024-12-31

**Authors:** Shiyang Zhang, Nicole Richards, Sibo Gao, Karen Fingerman

**Affiliations:** University of Texas at Austin, Austin, Texas, United States; The University of Texas Austin, Austin, Texas, United States; The University of Texas Austin, Austin, Texas, United States; The University of Texas Austin, Austin, Texas, United States

## Abstract

Older adults engage in in-person and remote forms of contact (phone, text). In-person contact may provide opportunities for sharing emotions and promoting well-being in ways that other modes of contact do not. This study examined expressing positive and negative emotions during different modes of social contact (in-person, phone), and the association between emotion expression and well-being throughout the day. Community-dwelling older adults aged 65 - 90 (N = 286, Mage = 73.95) from the Daily Experiences and Well-being Study completed ecological momentary assessments (EMA) during which they indicated their social contact as well as positive and negative mood every 3 hours for 5 to 6 days. Participants also wore Electronically Activated Recorders (EAR) to unobtrusively capture ambient sounds. Sound snippets that contained participants’ speaking (N = 30,311) were transcribed verbatim and processed using the Linguistic Inquiry and Word Count (LIWC) to obtain linguistic features pertaining to emotion expression. Multilevel models revealed that in-person and phone contact were both associated with an increased use of positive words (e.g., love, sweet), yet only in-person contact was associated with more words expressing negative feelings (e.g., worried, sad). Using both positive and negative words was associated with a better mood in the same 3 hours. No associations were found between either positive or negative words and negative mood. Findings suggest that older adults may benefit emotionally from expressing both positive and negative feelings during in-person contact. Interventions for older adults’ emotional well-being may focus on fostering emotionally meaningful social ties and providing in-person contact.

